# A comprehensive dataset on spatiotemporal variation of microbial plankton communities in the Baltic Sea

**DOI:** 10.1038/s41597-023-02825-5

**Published:** 2024-01-02

**Authors:** Meike A. C. Latz, Agneta Andersson, Sonia Brugel, Mikael Hedblom, Krzysztof T. Jurdzinski, Bengt Karlson, Markus Lindh, Jenny Lycken, Anders Torstensson, Anders F. Andersson

**Affiliations:** 1 KTH Royal Institute of Technology, Department of Gene Technology, Science for Life Laboratory, Stockholm, Sweden; 2 University of Copenhagen, Department of Plant and Environmental Sciences, Frederiksberg C, Denmark; 3 Umeå University, Department of Ecology and Environmental Sciences, Umeå, Sweden; 4 Umeå Marine Sciences Centre, Umeå University, SE-905 71 Hörnefors, Sweden; 5 Swedish Meteorological and Hydrological Institute, Community Planning Services - Oceanography, Västra Frölunda, Sweden; 6 Swedish Meteorological and Hydrological Institute, Oceanographic Research, Västra Frölunda, Sweden

**Keywords:** Microbial ecology, Marine biology, Environmental sciences

## Abstract

The Baltic Sea is one of the largest brackish water environments on earth and is characterised by pronounced physicochemical gradients and seasonal dynamics. Although the Baltic Sea has a long history of microscopy-based plankton monitoring, DNA-based metabarcoding has so far mainly been limited to individual transect cruises or time-series of single stations. Here we report a dataset covering spatiotemporal variation in prokaryotic and eukaryotic microbial communities and physicochemical parameters. Within 13-months between January 2019 and February 2020, 341 water samples were collected at 22 stations during monthly cruises along the salinity gradient. Both salinity and seasonality are strongly reflected in the data. Since the dataset was generated with both metabarcoding and microscopy-based methods, it provides unique opportunities for both technical and ecological analyses, and is a valuable biodiversity reference for future studies, in the prospect of climate change.

## Background & Summary

The Baltic Sea is one of the largest brackish water bodies on earth, a semi-enclosed continental sea with pronounced physicochemical gradients and seasonal dynamics. Particularly distinctive is the strong horizontal salinity gradient, created by freshwater discharge and limited water exchange with the North Sea^1,2^. The Baltic Sea is one of the most well-studied aquatic ecosystems in the world, with a long history of regular monitoring of physical, chemical, and biological variables^3^. Due to their central roles in biogeochemical cycles and their position at the base of the marine food chain, microbial plankton communities are specifically important in this ecosystem. In particular, since the Baltic Sea is strongly affected by anthropogenic eutrophication, which frequently and increasingly results in harmful algal blooms, as well as in large areas with hypoxic bottom waters due to microbial degradation of excess biomass^4–7^. Quantitative assessment of abundance and biodiversity of microbial plankton in the Baltic Sea is therefore not only central for environmental monitoring but does in addition provide a baseline biodiversity reference for future studies affected by ecosystem changes due to climate change. For that purpose, we created a systematic and comprehensive dataset to capture the variation in microbial communities along the salinity gradient of the Baltic Sea, Kattegat, and Skagerrak during a 13-month period, and complemented with data on physicochemical parameters from the same samples from the Swedish National Marine Monitoring Program.

**Fig. 1:**
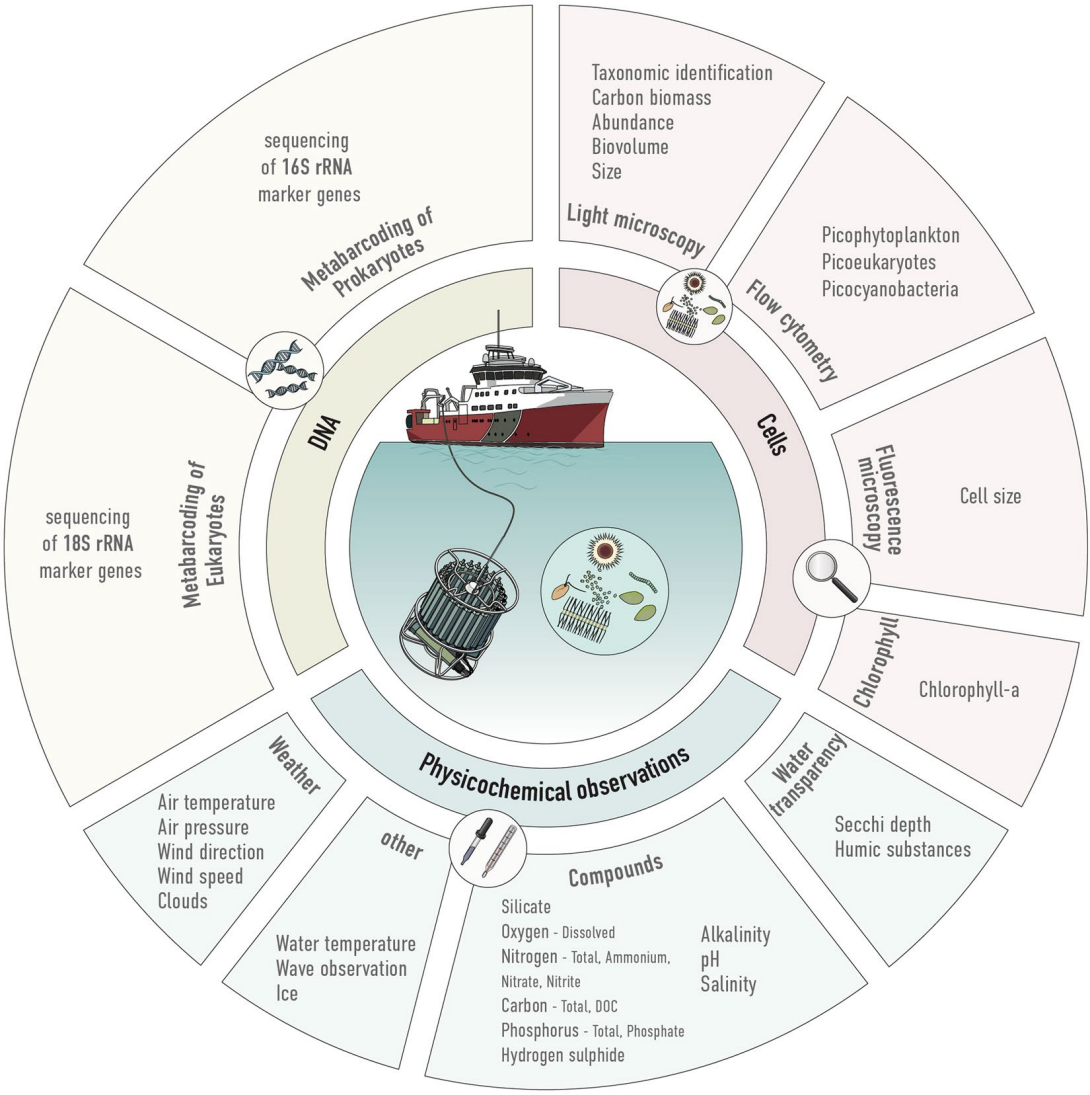
Overview of water analyses conducted, and parameters observed.

To obtain a comprehensive assessment of prokaryotic and eukaryotic microbial plankton diversity and the associated environmental conditions, we relied on both microscopy- and sequencing based methods complemented with extensive contextual parameters (Fig. 1). Both microscopy- and DNA sequencing-based methods can provide information on diversity and composition of microbial communities, and each have their advantages and limitations^8,9^. To date, marine monitoring of phyto- and microzooplankton is largely based on analyses using light microscopy, which cannot differentiate taxa with similar morphology and small sizes (less than ~5 µm) including all picoplankton 0.2–2 µm and some nanoplankton 2–20 µm. However, it can provide information on absolute abundance and biovolume of identifiable species. Metabarcoding is currently mainly used in research projects; it provides a qualitative and semi-quantitative assessment of the microbial community, can detect small and morphologically similar organisms and the data can be re-analysed at a later time when reference databases have improved. However, the short regions targeted by metabarcoding do not always provide species-level resolution and the data is not readily translatable to absolute abundances. Although spatial variation in Baltic Sea microbial communities have been reported for a few research cruises e.g.^10–14^, and temporal variation at single-stations e.g.^15,16^, no systematic large-scale survey of spatiotemporal variation has yet been reported with metabarcoding for this ecosystem.

**Fig. 2:**
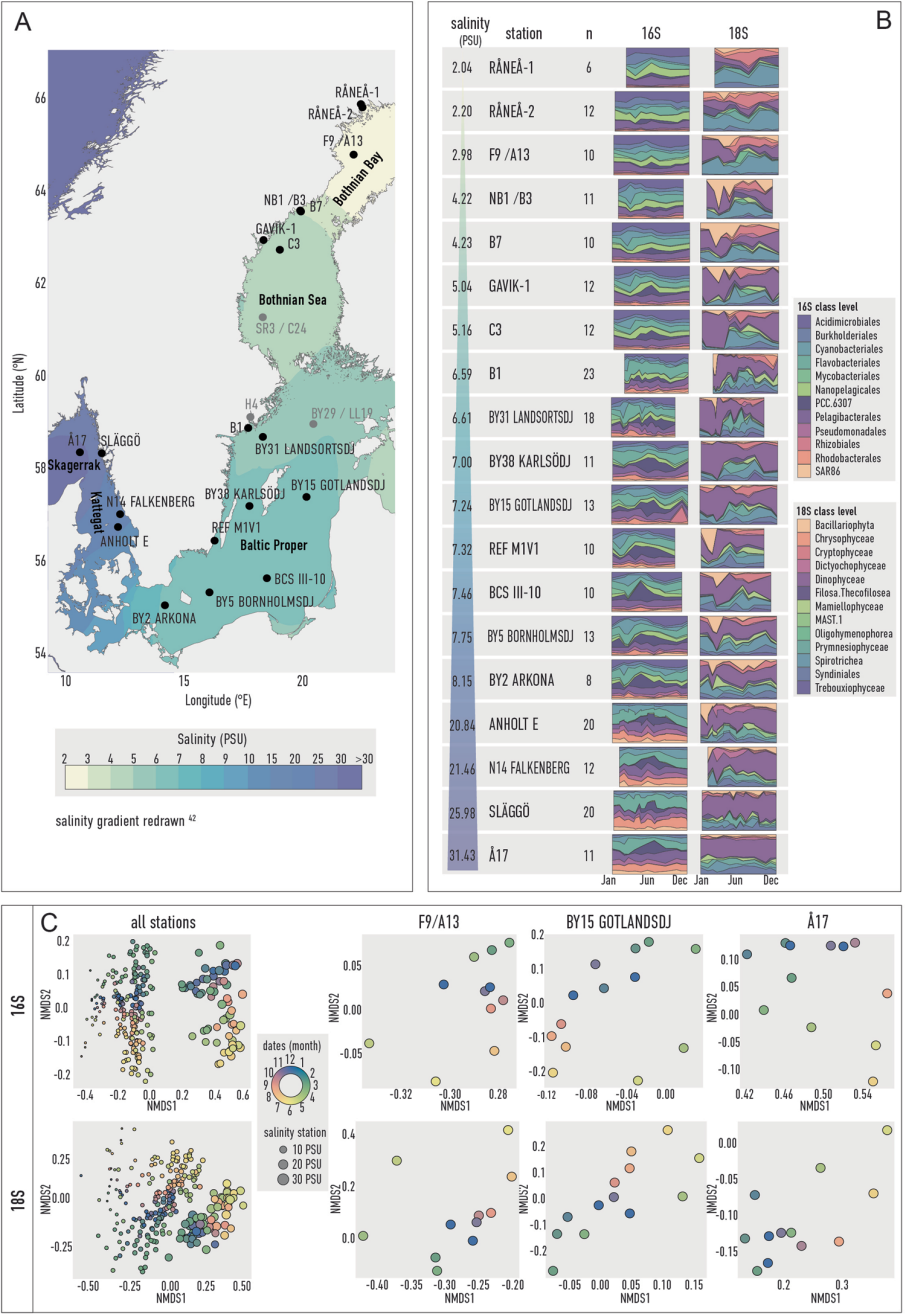
Overview of the sampled stations in the Baltic Sea, the Kattegat and the Skagerrak and general data structure based on 18S and 16S rRNA metabarcoding data. (**A**) Map of the Baltic Sea with its salinity gradient (redrawn from^46^) and the 22 sampled stations. Three of the stations (indicated with grey circles in the map) had less than five samples and were not included in the analyses presented in panels B and C. (**B**) Seasonal variation in relative abundance of prokaryotic (16S) and eukaryotic (18S) microbial plankton on class level at the individual stations. Mean salinity level (PSU) measured and number of samples taken (n) at each station are depicted. (**C**) NMDS plots of β-diversity measured by Bray-Curtis distance of 16S and 18S sequencing data; of all stations and three individual stations at the extremes and the middle of the salinity gradient. For plots of individual stations, data points are not sized by salinity.

Between January 2019 and February 2020, 278 transect-time course samples and 63 samples for protocol testing were collected from 22 stations in the Baltic Sea, Kattegat, and Skagerrak (Fig. 2a) (with ≥ 10 samples for 17 of the stations). The stations covered the salinity gradient of the Baltic Sea towards the opening to the Atlantic - through the Kattegat and Skagerrak (Fig. 2a), with average (over time) salinity ranging from 2 PSU in the Bothnian Bay to 31 PSU in the Skagerrak (Fig. 2a,b). We analysed the samples with 18S ribosomal RNA (rRNA) gene and 16S rRNA gene metabarcoding, to capture the eukaryotic and prokaryotic diversity, respectively. In the sequencing data, the influence of salinity and season on community composition is evident for both eukaryotic and prokaryotic plankton (Fig. 2b,c). The relative abundance of certain taxonomic groups varied along the salinity gradient, e.g. for the prokaryotic classes Rhodobacterales and SAR86 (orange shades) which were more abundant under high to moderate salinity. The communities diverged based on salinity and season in their β-diversity (Fig. 2c); the sampling gap in the salinity gradient separating the samples into two clusters.

Complementary analyses on the same samples were conducted by the Swedish National Marine Monitoring Program: Microscopy analyses were performed on samples preserved in Lugol’s solution^17^ for phytoplankton identification, estimation of cell volumes and cell counts. In addition, samples from nine stations were quantified for phototrophic picoplankton using flow cytometry and fluorescence microscopy. Additionally, chlorophyll-*a* content was assessed in the water as a proxy for total phytoplankton biomass and a range of physicochemical parameters were measured (Fig. 1).

The various datasets presented here are of interest for an array of studies encompassing for example microbial ecology and methodological assessments. It can be used for investigating microbial community structure in the Baltic Sea and the influence of environmental parameters, distribution of organisms and harmful algae blooms, co-occurrence patterns, as well as for comparing microscopy *vs*. metabarcoding methods for plankton monitoring.

## Methods

### Sampling

In total, 341 water samples were collected (278 transect-time course samples and 63 samples for protocol testing) from January 2019 to February 2020 at 22 stations in the Baltic Sea, Kattegat, and Skagerrak (Fig. 2a), during monthly/bi-weekly sampling cruises. The 278 transect-time course samples were collected during cruises that were part of the Swedish National Marine Monitoring Program, implemented by the Swedish Meteorological and Hydrological Institute (SMHI; Skagerrak, Kattegat and Baltic Proper), Umeå University (UmU; Bothnian Sea and Bothnian Bay) and Stockholm University (SU; stations BY1 and BY31 in the Baltic Proper) on different research vessels specified for each sample by the vessel’s ICES (International Council for the Exploration of the Seas) platform code. Samples were collected and physicochemical parameters measured using a Conductivity Temperature Depth (CTD) profiling instrument (model SBE 911plus/SBE19+, Sea Bird Electronics Inc., Bellevue, Washington, USA) deployed on a rosette (model SBE32). Water for the microbial analyses was sampled with a depth-integrating hose covering the depth of 0–10 m. At stations B1 and BY31 the depth covered was 0–20 m and station RÅNEÅ-1 0-5 m. Physicochemical parameters were measured at 0, 5, and 10 m depth. Samples for these measurements were collected using Niskin bottles. For testing the sampling and sample storage protocol, a total of 63 samples were taken to evaluate the effect of sample filtering volume (10, 100, 200 and 500 mL) and filter storage temperature (−20 °C and −80 °C). Over three sampling occasions (dates: 2019-05-06, 2019-08-06, 2019-10-07) at station SLÄGGÖ, 57 samples were collected to test filtering volume and 6 for testing storage temperature. Additionally, replicates were taken on six sampling occasions (three occasions with two replicates, and three occasions with five replicates, these samples are not counted into the total count). Altogether, the data were collected at 263 unique sampling occasions.

### DNA extraction and sequencing

For DNA analyses, 500 mL of seawater were filtered onto a 47 mm membrane filter of 0.22 µm pore size (GSWP04700, MilliporeSigma, Burlington, MA, USA) using a filter funnel with a < 270 mbar/200 mm Hg vacuum. The filtration was initiated within one hour after sampling, and the filtration time was kept below one hour or otherwise noted. Subsequently, the filters were rolled into a 5 mL cryotube, flash-frozen in liquid nitrogen and stored at −20 °C until further processed. DNA was extracted^18^, libraries prepared for metabarcoding of 16S rRNA^11^ and 18S rRNA^19,20^, and sequenced on Illumina MiSeq flow cells. This resulted in an average output of 130 thousand paired-end read pairs per sample (0.171 for 16S and 0.095 for 18S).

DNA extraction from filters was performed using the ZymoBIOMICS™ DNA Miniprep Kit (Zymo Research Corp, Irvine, CA, USA) following the manufacturer’s instructions with a few modifications^18^: After adding the lysis buffer to the filter (and before bead-beating), 10 µL of spike-in DNA were added to each sample (described in the next section). The bead beating conditions were optimised to 10 min and for elution of DNA from the column, 50 µL were used instead of 100 µL. The concentration and quality of the DNA was assessed using the Qubit™ dsDNA HS Assay Kit on a Qubit Fluorometer (ThermoFischer, Waltham, MA, USA) and an Agilent DNA High Sensitivity Kit on a 2100 Bioanalyzer instrument (Agilent Technologies, Santa Clara, CA, USA). DNA extraction of the samples collected by SMHI and SU was conducted at SMHI, and of the samples collected by UmU at UmU. Sequencing libraries for 18S rRNA metabarcoding targeting the hypervariable V4 region of the eukaryotic 18S rRNA gene were prepared using the primers V4F CCAGCASCYGCGGTAATTCC and V4RB ACTTTCGTTCTTGATYRR^19^ with the simplified PCR protocol described in^20^. Libraries for 16S rRNA metabarcoding targeting the hypervariable V3-V4 regions of the bacterial 16S rRNA gene were prepared following the protocol^21,22^ with the primers 341 F CCTACGGGNGGCWGCAG and 805 R GACTACHVGGGTATCTAATCC^11^. The primers were supplemented with 5′-end Illumina sequence adapters (forward: ACACTCTTTCCCTACACGACGCTCTTCCGATCT-3′, reverse: 5′-GTGACTGGAGTTCAGACGTGTGCTCTTCCGATCT) and ordered from IDT DNA (IA, US) at 100 μM in TE buffer. To increase the complexity of the libraries, phased primers^22,23^ were used for the 18S forward primer, with equal proportions of primers having ATG, TG, G, or no base inserted between the adapter sequence and the target-binding region. For 16S, phasing was used on both primers, with CTAGAGT, TAGAGT, etc for the forward and ACTACTG, CTACTG, etc for the reverse. The PCR reactions were carried out with the KAPA HiFi HotStart ReadyMix PCR Kit (Kapa Biosystems, MA, USA), according to the manufacturer’s instructions, with the final 25 µL reaction mix containing 1x Kapa HiFi HotStart ReadyMix, 0.3 μM of each primer, and 5 ng template DNA for 18S library preparation and 1 ng for 16S. For 18S rRNA amplification the PCR conditions were 95 °C for 3 min, 20 cycles of 98 °C for 20 s, 52 °C for 15 s and 72 °C for 15 s, followed by a final elongation step of 72 °C for 2 min. For 16S rRNA amplification the following PCR conditions were used: 98 °C for 2 min, 20 cycles of 98 °C for 20 s, 54 °C for 20 s and 72 °C for 15 s, followed by a final elongation step of 72 °C for 2 min. The PCR product was cleaned with magnetic beads using the MagSi-NGS PREP Plus Kit (MDKT00010075, magtivio BV., Nuth, the Netherlands), indexed through a second PCR with Kapa HiFi HotStart ReadyMix, equimolar pooling and sequencing on three MiSeq lanes (Illumina Inc, San Diego, CA, US) for 18S and 16S rRNA metabarcoding, respectively. The PCR conditions for indexing were 95 °C for 2 min, 8 cycles of 98 °C for 20 s, 55 °C for 30 s and 72 °C for 30 s, followed by a final elongation step of 72 °C for 2 min. The Adapterama indexing scheme was used^24,25^, using unique forward and reverse indices for every sample sequenced together. Library preparation and sequencing were conducted by the Swedish National Genomics Infrastructure (NGI) at SciLifeLab (Solna, Sweden).

A few individual libraries yielded no sequencing data; samples that neither generated 16S nor 18S rRNA metabarcoding data were removed from the dataset. From one sample collected from station SR3/C24 on 2019-06-10 we obtained 16S data only, and one sample from station BY2 ARKONA collected 2019-06-10 18S data only. From the 59 samples taken for volume testing, several did not yield sequencing data for either 16S and 18S rRNA metabarcoding. For seven we obtained only 16S data (volumes 10–500 mL), and for nine samples we obtained only 18S data (volumes 10–200 mL), and for two 10 mL samples we obtained no sequencing data.

### Spike-in DNA

To facilitate estimation of absolute abundances of individual barcode sequences (ASVs) in the samples, we added known amounts of synthetic spike-in DNA during the DNA extractions. We designed individual spike-in DNA sequences for 18S (“EnvGen_18S_spike”) and 16S (“EnvGen_16S_spike”) rRNA metabarcoding^18,26^, with random nucleotide sequences of the same GC content as the consensus of the Protist Ribosomal Reference (PR2) PR2 database^27^ for 18S, and of the 16S rRNA gene of *Escherichia coli* strain NR_024570.1 for 16S. Primer binding sites were inserted in the sequences, separated by distances based on the 18S rRNA consensus of PR2^27^ and on the 16S rRNA gene of *E. coli* strain NR_024570.1 (amplicon length of 408 bp for 18S, and 428 bp for 16S) (the sequences of the spike-ins are provided in^26^). The oligonucleotides were ordered as dry pellets (Twist Bioscience, San Francisco, CA, USA), diluted to the desired concentration with a dilution series, and aliquots stored at −80 °C to avoid degradation of DNA in repeated freeze-thaw cycles. The amount of spike-in DNA to be added was estimated based on amounts of DNA extracted from Baltic Sea water samples in a previous publication^13^, combined with data on proportions of sequence reads in Baltic Sea shotgun metagenomics data^28^ that encode 18S and 16S rRNA (that we estimated with Metaxa2^29^). For the DNA extractions conducted at UmU, 7 pg of 18S and 2 pg of 16S spike-in DNA was added to each DNA extraction. For the DNA extractions conducted at SMHI, 10 pg of 18S and 2.3 pg of 16S spike-in DNA was added to each DNA extraction. In the final dataset, the average spike-in percentages of quality-filtered reads were 6.96% and 1.51% for 18S and 16S, respectively.

### Processing of sequencing data

Initially, sequences of phased primers were removed from the reads using a snakemake pipeline^30^ that utilises cutadapt^31^. The pipeline conducts the following steps: removes read-pairs containing Illumina adapters, removes read-pairs that do not contain the expected primer sequences in the 5′ ends of the reads and removes the primer sequences from the remaining reads, removes read-pairs that contain primer sequences anywhere else on the reads, trims reads to fixed lengths. Further analyses of sequencing data and plotting of the data was performed in R version 4.0.3 using the packages ‘DADA2’^32^ version 1.18.0, ‘vegan’^33^ version 2.5–7, and ‘ggplot2’^34^ version 3.4.0. The median sequencing depth was 0.13 M read pairs per sample with >80% of reads of a quality score >30 for both 18S and 16S rRNA amplicons. The package ‘DADA2’ was used to infer biological sequence variants from amplicon reads; the individual sequencing runs were processed separately and merged after obtaining the sequence tables. Low-quality reads were filtered out. The remaining reads were denoised (using pool = F) and forward and reverse reads merged. This resulted in 10,293 amplicon sequence variants (ASVs) for 18S rRNA and 40,369 ASVs for 16S rRNA. Taxonomy of the ASVs was inferred with ‘assignTaxonomy’ using PR2^27^ version 4.14.0 as a training set for 18S rRNA amplicons and a curated version^35^ of the 16S sequences of Genome Taxonomy Database (GTDB; version R06-RS202-1)^36^ for 16S rRNA amplicons. For the analyses of the data presented in this publication, one 18S sample with unusually high read number was removed, and from the replicated samples, one was randomly chosen. The ASVs from the spike-in DNA sequences were identified and removed from the ASV table, sequences assigned to Metazoa were also removed. Finally, ASV counts were rarefied to the same total counts per sample with the function ‘rrarefy’ from the ‘vegan’ package version 2.5–7 to ~44,000 for 16S and ~8,000 for 18S. Using cumulative sum scaling^37^ normalisation generated similar beta-diversity patterns, and the Bray-Curtis distances generated with the two approaches were highly correlated (r = 0.96 for 16S and r = 0.92 for 18S).

### Microscopy and flow cytometry

Light microscopy was performed for counting and determining cell size of phytoplankton as previously described^17^ and further described^38–40^. In short, after sampling with an integrated hose (0–10 m depth - 0–20 m or 0–5 m for some stations) and mixing of the water, subsamples were fixed immediately in acidic Lugol’s iodine solution, and settled in a sedimentation chamber. The fixed samples were later counted under an inverted microscope; a detailed protocol can be accessed under Annex C-6 of the HELCOM-COMBINE manual^41^. Briefly, Lugol fixed samples (10 or 25 ml depending on phytoplankton abundance) were settled in sedimentation chambers (for 10 to 24 hours depending on the volume) and counted in an inverted microscope using phase contrast or differential interference contrast. A total of minimum 188 cells was counted and a minimum of 33 units of the most abundant taxa. The HELCOM-PEG list of species, biovolumes and carbon content^39,42^ was used as reference for taxonomy and biovolumes.

Phototrophic picoplankton was counted by epifluorescence microscopy. Samples were preserved in formaldehyde (4% final concentration) and stored at +4 °C. Of the preserved sample, 5 to 20 mL were filtered onto black 0.2 µm polycarbonate filter (Whatman Cytiva, USA) with paper filter (GF/C Whatman Cytiva, USA) as support and at maximum vacuum of 100 mm Hg. Filters were mounted on slides with immersion oil, later frozen at −20 °C and analysed within a year. Picoplankton samples were analysed at 1000x using an inverted epifluorescence microscope. Picocyanobacteria were analysed using a green excitation light (excitation 510–560 nm band pass, emission at 590 nm) and eukaryotic picophytoplankton with a blue excitation light (excitation 450–490 nm band pass, emission at 515 nm). All picoplankton in a field of view of 100 × 100 µm were counted; at least 300 cells or 30 fields of view were counted.

Flow cytometry of phototrophic picoplankton was performed on samples from the stations B7, C3, F9/A13, GA1/GAVIK-1, NB1/B3, RA1/RÅNEÅ-1, RA2/RÅNEÅ-2, SLÄGGÖ, and SR3/C24. For flow cytometry, the samples were preserved in 0.1% glutaraldehyde (final concentration) and stored at −20 °C. Frozen samples were quickly thawed in a 30 °C water bath before analysis with a BD FACSVerse™ flow cytometer (BD Biosciences, Stockholm, Sweden), equipped with a blue (488 nm) and red laser (640 nm). Picophytoplankton samples were analysed at a flow rate of 120 μl min^−1^ for 2 min with 3 µm microspheres (Fluoresbrite plain YG, Polysciences, PA, Warrington) as internal standards. Picoeukaryotes and phycoerythrin-rich (PE) and phycocyanin-rich (PC) picocyanobacteria were differentiated based on their red (700 ± 27 nm for chlorophyll and 660 ± 5 nm for phycocyanin) and orange fluorescence (586 ± 21 nm for phycoerythrin).

### Measurement of physical and chemical parameters and chlorophyll

Observations of physical and chemical parameters and chlorophyll-*a* were conducted according to the HELCOM-COMBINE manual^41^. Physical parameters measured included wind direction and speed, air temperature and pressure, clouds, wave observation, ice, salinity, water temperature, pressure, conductivity, and Secchi depth (water transparency). The sea water chemistry was also analysed (instruments specified differed between the institutes conducting the sampling, the instruments specified were used at SMHI): Alkalinity (total alkalinity) was determined by potentiometric titration (Metrohm 888 Titrando with LL Aquatrode Plus Pt1000 electrode), and pH measured using a Thermo Scientific Orion 8102BNUWP ROSS Ultra electrode. Inorganic nutrients including silicate (SiO_3_-Si), phosphate (PO_4_-P), total phosphorus (Tot-P), nitrate (NO_3_-N), nitrite (NO_2_-N), nitrite + nitrate (NO_2_ + NO_3_-N) and total nitrogen (Tot-N) were determined using an OI Analytical Flow Solution IV colorimetric analyser with ER detector. Ammonium (NH_4_-N) was detected using an OI Analytical Flow Solution IV with a D-Star Instruments DFL-10 Fluorescence detector. Dissolved oxygen (O_2_) was determined by potentiometric titration (Winkler method) using a Metrohm 888 Titrando with Micro Pt Titrode. Hydrogen sulphide (H_2_S) was measured colourimetrically using a Hitachi U-1900 spectrophotometer. Dissolved organic carbon (DOC) and total organic carbon (TOC) were analysed by Eurofins Environment, Lidköping, Sweden, using the non-dispersive infrared gas analyser technique. Humic substances were analysed using a Hitachi F-2710 fluorescence spectrophotometer.

Water samples for chlorophyll pigment analysis were sampled with an integrated hose or CTD-rosette bottles and immediately filtered onto glass microfiber filters (GF/F, Whatman Cytiva, USA). Samples were extracted with ≥96% ethanol and stored at ‒20 °C until analysis with a spectrofluorometer, further described in the HELCOM COMBINE manual Annex C-4 39.

### Contextual data

All contextual data (physical and chemical, chlorophyll-a, light- and fluorescence microscopy, and flow cytometry data) can be accessed through our figshare repository^26^ (10.17044/scilifelab.20751373). It can also be downloaded from the Swedish National Oceanographic Data Centre using an R script provided in the figshare repository (also available here: https://github.com/anderstorstensson/sharkdata-r-download).

## Data Records

The raw sequencing data generated in this study are available at the European Nucleotide Archive (ENA)^43^ under the study accession number https://identifiers.org/ena.embl:PRJEB55296 (2023). Processed sequencing data (ASV sequences with taxonomic annotations and counts in samples) are available at our figshare repository^26^ (10.17044/scilifelab.20751373), along with the contextual, physicochemical, and microscopy data, and sequences of synthetic spike-ins. All physicochemical data can also be downloaded through SHARKweb as described above; detailed instructions on accessing specific parts of the data are available in the figshare repository^26^. Processed sequencing data (ASVs of 18S and 16S rRNA gene metabarcoding) can also be accessed and viewed interactively through the ASV-portal^44^ https://asv-portal.biodiversitydata.se at the Swedish Biodiversity Infrastructure (SBDI) as well as through the Global Biodiversity Information Facility (GBIF) using 10.15468/vrxhxe for the 16S and 10.15468/cwjstg for the 18S data.

## Technical Validation

**Fig. 3:**
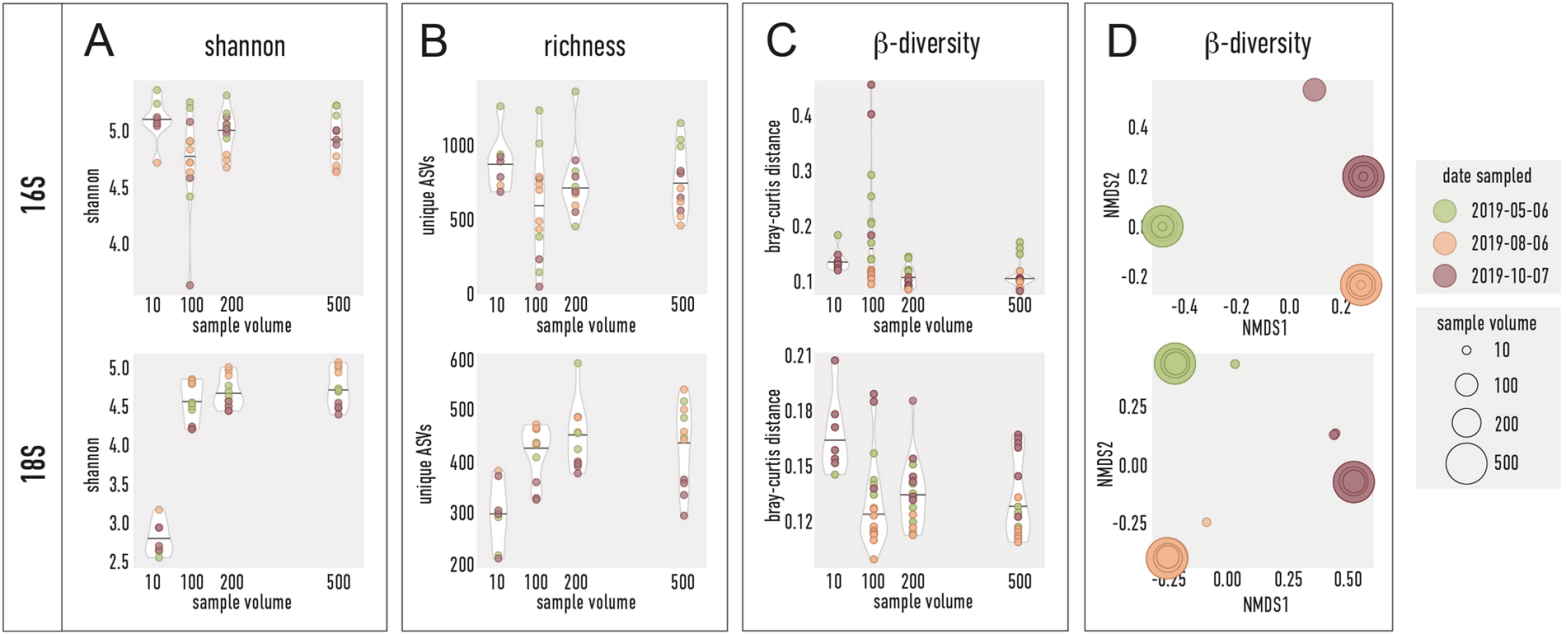
Evaluation of water sample volumes required for metabarcoding. Samples from three sampling occasions (circle colour) and taken in different sample volumes of 10–500 ml (circle size in panel D) were taken in five replicates. (**A**) α-diversity measured by Shannon index, (**B**) α-diversity as richness measured by number of unique ASVs, (**C**) violin plots of β-diversity between replicates and (**D**) NMDS plots of β-diversity between all samples. β-diversity was measured with Bray-Curtis distance.

Many of the procedures for sampling and measurement of environmental parameters are optimised and routinely performed within the Swedish National Marine Monitoring Program, commissioned by the Swedish Agency for Marine and Water Management, and the countries surrounding the Baltic Sea (HELCOM)^41^. In this study, we performed technical validations of the protocols for sampling, sample storage and processing, sequencing library preparation, and quality of the data. We compared different sample filtration volumes (10, 100, 200, 500 ml) taken in five replicates on three sampling occasions at the SLÄGGÖ station (Fig. 3) to validate that 500 ml was sufficient to cover the microbial diversity. Both α-diversity measured by Shannon index and richness appeared to reach a plateau at around 200 ml sample volume (Fig. 3a,b), and the variation between the replicates decreased with sample volume up to this point (coloured dots within the violin plots). We further compared the influence of sample storage at −20 °C *vs*. −80 °C on three replicates for a three-months storage period (data not shown) with no significant differences in Shannon α-diversity (Wilcoxon rank sum exact test, p-value 1 and 0.1 for 16S and 18S, respectively) but ANOSIM analysis indicated an effect on community composition, although not significant (ANOSIM analysis on Bray-Curtis distances, R-value: 0.67 and 1 and p-value 0.1 and 0.1, for 16S and 18S, respectively). Blanks (filters without sample, but with spike-in DNA added during extraction) were PCR amplified with the 16S and 18S primers and sequenced to detect contamination sources during the DNA extraction procedure. In the three blank samples, the spike-in sequences corresponded to more than 99.7% of all counts in all cases for 18S rRNA metabarcoding; for 16S it was 97.0%, 95.3%, and 93.4%, respectively.

We tested the influence of two DNA extraction kits (Qiagen DNeasy PowerWater Kit and ZymoBIOMICS™ DNA Miniprep Kit, the latter used for the other samples of this study) on the Shannon diversity obtained from 16S and 18S rRNA metabarcoding on six water samples from two stations (N14 FALKENBERG and HANÖBUKTEN) and did not find a significant difference in obtained α-diversity between the kits (Shannon α-diversity, Wilcoxon rank sum exact test, p-value = 0.96 and 0.10, for 16S and 18S, respectively) while community composition was affected (ANOSIM analysis on Bray-Curtis distances, R-value: 0.58 and 0.20, P-value 0.003 and 0.024, for 16S and 18S, respectively) (data not shown, available upon request). The aim of the kit comparison was to investigate if the data is comparable with data from previous sampling efforts, where the Qiagen kit was used^13^. This calls for some caution when comparing datasets generated using these two kits.

We evaluated primers most suitable for metabarcoding of eukaryotic plankton in a previously published study^20^. In order to improve the sequencing quality, we used phased primers to increase the complexity of amplicon sequencing libraries^22,45^; for the 18S primer pair phasing was only used in the forward primer.

The sequencing reads were processed following the DADA2 pipeline^32^ to trim and filter low quality reads, infer true sequence variants taking the error rates of the sequencing run into consideration, and removing chimeras from the dataset. The sequencing data validity is also confirmed by the fact that the salinity gradient and seasonality is reflected (Fig. 2b,c) as shown in previous studies^11,13,28^.

## Usage Notes

The spike-in reads are part of raw data deposited on ENA (PRJEB55296) but not of the data that can be accessed through SBDI, GBIF and SHARKweb.

## Data Availability

Code for sequence data processing and for reproducing the graphs of this paper is available through our figshare repository^26^ (https://figshare.com/s/b2962b2174747c6bc869).
